# Effects of *Phlomis umbrosa* Root on Longitudinal Bone Growth Rate in Adolescent Female Rats

**DOI:** 10.3390/molecules21040461

**Published:** 2016-04-07

**Authors:** Donghun Lee, Young-Sik Kim, Jungbin Song, Hyun Soo Kim, Hyun Jung Lee, Hailing Guo, Hocheol Kim

**Affiliations:** Department of Herbal Pharmacology, College of Korean Medicine, Kyung Hee University, Seoul 130-701, Korea; allstart2925@naver.com (D.L.); yjbsik@naver.com (Y.-S.K.); jbsong0527@gmail.com (J.S.); lovelyradix@hanmail.net (H.S.K.); lhjung91@hanmail.net (H.J.L.); guohailing1026@gmail.com (H.G.)

**Keywords:** *Phlomis umbrosa*, bone growth rate, IGF-1, growth plate

## Abstract

This study aimed to investigate the effects of *Phlomis umbrosa* root on bone growth and growth mediators in rats. Female adolescent rats were administered *P. umbrosa* extract, recombinant human growth hormone or vehicle for 10 days. Tetracycline was injected intraperitoneally to produce a glowing fluorescence band on the newly formed bone on day 8, and 5-bromo-2′-deoxyuridine was injected to label proliferating chondrocytes on days 8–10. To assess possible endocrine or autocrine/paracrine mechanisms, we evaluated insulin-like growth factor-1 (IGF-1), insulin-like growth factor binding protein-3 (IGFBP-3) or bone morphogenetic protein-2 (BMP-2) in response to *P. umbrosa* administration in either growth plate or serum. Oral administration of *P. umbrosa* significantly increased longitudinal bone growth rate, height of hypertrophic zone and chondrocyte proliferation of the proximal tibial growth plate. *P. umbrosa* also increased serum IGFBP-3 levels and upregulated the expressions of IGF-1 and BMP-2 in growth plate. In conclusion, *P. umbrosa* increases longitudinal bone growth rate by stimulating proliferation and hypertrophy of chondrocyte with the increment of circulating IGFBP-3. Regarding the immunohistochemical study, the effect of *P. umbrosa* may also be attributable to upregulation of local IGF-1 and BMP-2 expressions in the growth plate, which can be considered as a GH dependent autocrine/paracrine pathway.

## 1. Introduction

Short stature is defined as the height of an individual more than two standard deviation score (SDS) below the average height for an age, sex, and population group [1]. Short children might have a high frequency of psychosocial problems such as lower self-esteem, social immaturity, or being bullied [2,3,4,5]. People with shorter height are reported to have a lower health-related quality of life even if they do not fit into the definition of short stature [6]. People with shorter height also have higher morbidity of coronary heart diseases, perhaps because of the smaller diameter of their blood vessels [7,8].

Short stature caused by certain diseases, including growth hormone (GH) deficiency, accounts for 20% of all short children, but the remaining 80% are not determined, so-called idiopathic short stature (ISS) [1]. In case of GH deficiency, average increase in final height attributable to GH therapy is about 30 cm compared with predicted adult height [9,10]. In the case of ISS, the US FDA approved GH therapy in ISS children shorter than −2.25 SDS in 2003 based on the evidence derived from two clinical studies [11,12], nevertheless, the average increase in final height attributable to GH therapy in children with ISS is just 3.5–7.5 cm (4–7 years) [11,13,14,15,16]. In this case of ISS, the effect remains controversial because the estimated cost of GH therapy for adult height gain is about 10,000–20,000 dollars/cm [1,15]. Moreover, children’s pain due to daily injection and abuse of human GH to increase the height of children who are already of normal height are also considered controversial [17,18]. 

For these reasons, alternative oral growth stimulators with relatively low cost are currently being studied. Up to now, aromatase inhibitors, arginine, which is an essential amino acid for children, and zinc, which is an essential micronutrient, have been studied to stimulate bone growth or GH secretion. Aromatase inhibitors can delay puberty, thereby increasing the growth rate of male children in clinical trials, but their safety of delayed puberty has not yet been demonstrated [19]. Arginine increases GH secretion when it is administered intravenously; nevertheless, this effect only lasts for a few hours and it is often ineffective when administered orally [20]. Supplementation of zinc increases longitudinal growth rate in the children with zinc-deficiency, but it is not effective in cases of healthy normal children [21,22].

In accordance with traditional Korean medicinal theory that height growth of children occurs under the influence of innate *qi* and acquired *qi*, medicinal herbs which are used to tonify innate *qi* or acquired *qi* were selected from the *Dongeuibogam*. We have screened natural products to develop the safe growth stimulators with health benefits by an established *in vivo* model which can evaluate the bone growth rate in adolescent rats using tetracycline [23]. The root of *Phlomis umbrosa* Turcz. (Lamiaceae) was identified as one of the most effective herbs in this screening process.

*P. umbrosa*, also known as *Hansokdan*, *Sokdan*, or *Caosu* was used as *Xu Duan*, which is widely used to strengthen the muscles and bones and treat bone fractures, until the Song dynasty in China. *P. umbrosa* was also used as *Sokdan* (*Xu Duan*) in Korea, including *Dongeuibogam*, until the twentieth century [8,24,25]. Among the Tujia people of China, *P. umbrosa* root has been used to treat rheumatic disease, bone fractures and bleeding. *P. umbrosa* was reported to have anti-allergic, anti-inflammatory, and anti-nociceptive effects [26,27,28]. *P. umbrosa* contains iridoids and phenylethylalcohol, and the most prevalent iridoids are 8-*O*-acetylshanzhiside methyl ester, lamiide and shanzhiside methyl ester, known to be responsible for its diverse biological activities [29,30]. The compound 8-*O*-acetylshanzhiside methyl ester has been reported to attenuate cerebral ischemia and myocardial ischemia injury through anti-inflammatory mechanism, and improve angiogenesis and functional recovery after cerebral ischemia [31,32,33].

Height growth is the consequence of proliferation and hypertrophy of chondrocytes in the growth plates, which is called endochondral ossification, and this is caused by direct stimulation of GH or circulating insulin-like growth factor-1 (IGF-1) [34,35]. To increase the adult height, the growth rate should be increased without affecting growth period and the rate is determined by the rate of chondrocyte proliferation, subsequent differentiation and mineralization in certain period of time [36].

To observe the daily bone growth rate, tetracycline was used in this study as an intravital marker to stain newly formed bone in the growth plate, [37]. We have also analyzed the effects of *P. umbrosa* on zonal height and chondrocyte proliferation in growth plate of proximal tibia. To assess the possible endocrine/paracrine mechanism whereby *P. umbrosa* exerts its growth-promoting effects, we evaluated the IGF-1, insulin-like growth factor binding protein-3 (IGFBP-3) or bone morphogenetic protein-2 (BMP-2) in response to *P. umbrosa* administration in either growth plate or serum.

## 2. Results

### 2.1. HPLC Analysis of P. umbrosa Extract

**Figure 1:**
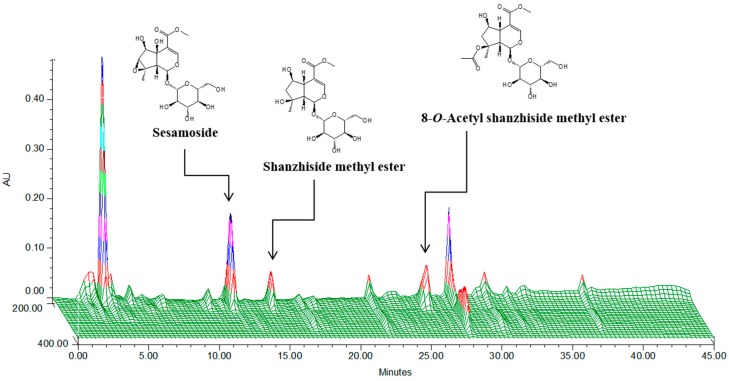
Three-dimensional high-performance liquid chromatogram for standardization of *P. umbrosa*.

*P. umbrosa* extract was standardized to contain 6.62 mg/g of shanzhiside methyl ester. A three dimensional HPLC chromatogram and the structures of the constituent compounds are shown in Figure 1.

### 2.2. Effect on Longitudinal Bone Growth Rate

**Figure 2:**
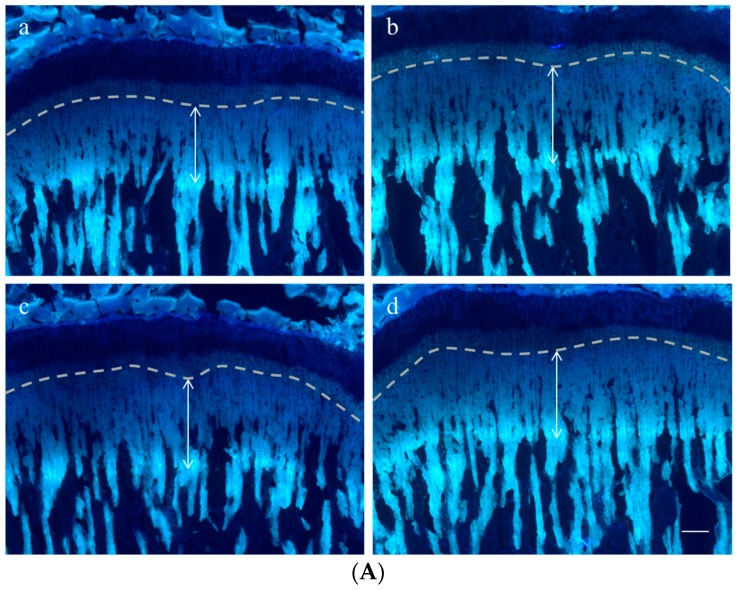

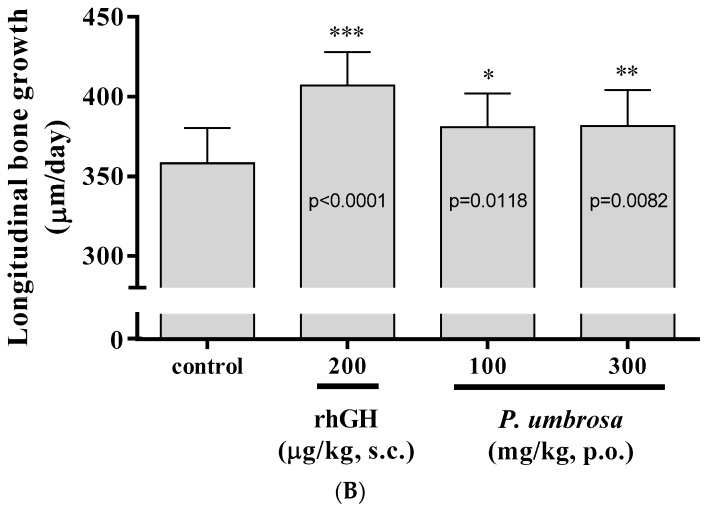
(**A**) Representative fluorescence photomicrographs of sagittal sections of the proximal tibial growth plate in rats. The double-headed arrow shows the distance between the chondro-osseous junction within the growth plate and the proximal endpoint of the tetracycline label which indicates the length of bone growth in proximal tibial growth plate during 72 h period. (**a**) vehicle treated control group; (**b**) rhGH 200 μg/kg (s.c.) treated group; (**c**) *P. umbrosa* 100 mg/kg (p.o.) treated group; (**d**) *P. umbrosa* 300 mg/kg (p.o.) treated group. The scale bar is 200 μm; (**B**) Effects of *P. umbrosa* on longitudinal bone growth rate in proximal tibial growth plate. Each value is the mean ± SD. The number of animals is 14–21 per group; * *p* < 0.05, ** *p* < 0.01 and *** *p* < 0.001 *vs.* control (one-way ANOVA, Bonferroni test).

To evaluate the effect of *P. umbrosa* on longitudinal bone growth rate, tetracycline was used as a fluorescent marker to label the newly formed bone under the growth plate of the proximal tibia. The double-headed arrow indicates the length of bone growth in the proximal tibial growth plate during a 72 h period (Figure 2A). The distance was significantly increased by oral administration of *P. umbrosa* at doses of 100 or 300 mg/kg compared with the control group. Figure 2B shows the numerical values of longitudinal bone growth rate. Longitudinal bone growth rate in the control group was 358.2 ± 22.0 μm/day, and in rhGH treated group was 406.9 ± 21.1 μm/day. Oral administration of 100 and 300 mg/kg *P. umbrosa* significantly increased the longitudinal bone growth rate exhibiting 380.9 ± 21.1, and 381.5 ± 22.5 μm/day compared with the control group, respectively.

### 2.3. Effect on Growth Plate Height

**Figure 3:**
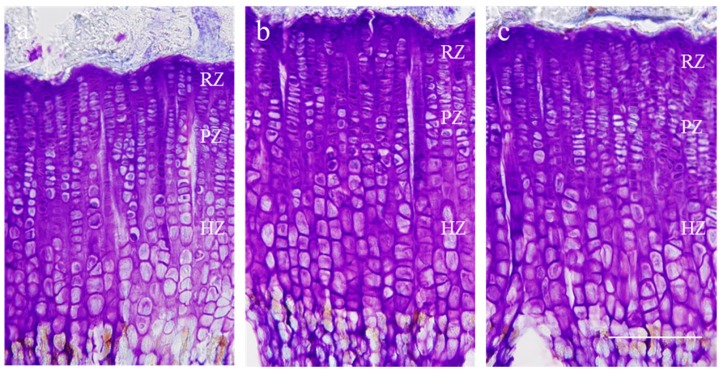
Representative photomicrographs of cresyl violet-stained chondrocytes of the proximal tibial growth plate in rats. (**a**) vehicle treated control group; (**b**) rhGH 200 μg/kg (s.c.) treated group; (**c**) *P. umbrosa* 300 mg/kg (p.o.) treated group, RZ: resting zone, PZ: proliferative zone, HZ: hypertrophic zone. The scale bar is 200 μm.

Proximal tibial growth plate height was measured using CV staining. Typical images of sections stained with CV are shown in Figure 3. The overall height of growth plate was 453.8 ± 28.6 μm in the control group and in the rhGH treated group it was 536.9 ± 29.5 μm. Oral administration of 300 mg/kg *P. umbrosa* significantly increased growth plate height, reaching 508.6 ± 28.9 μm compared with the control group. The heights of the hypertrophic zones, especially, were significantly increased in the rhGH and *P. umbrosa* groups (Table 1) compared to the control groups.

### 2.4. Effect on Chondrocyte Proliferation

**Figure 4:**
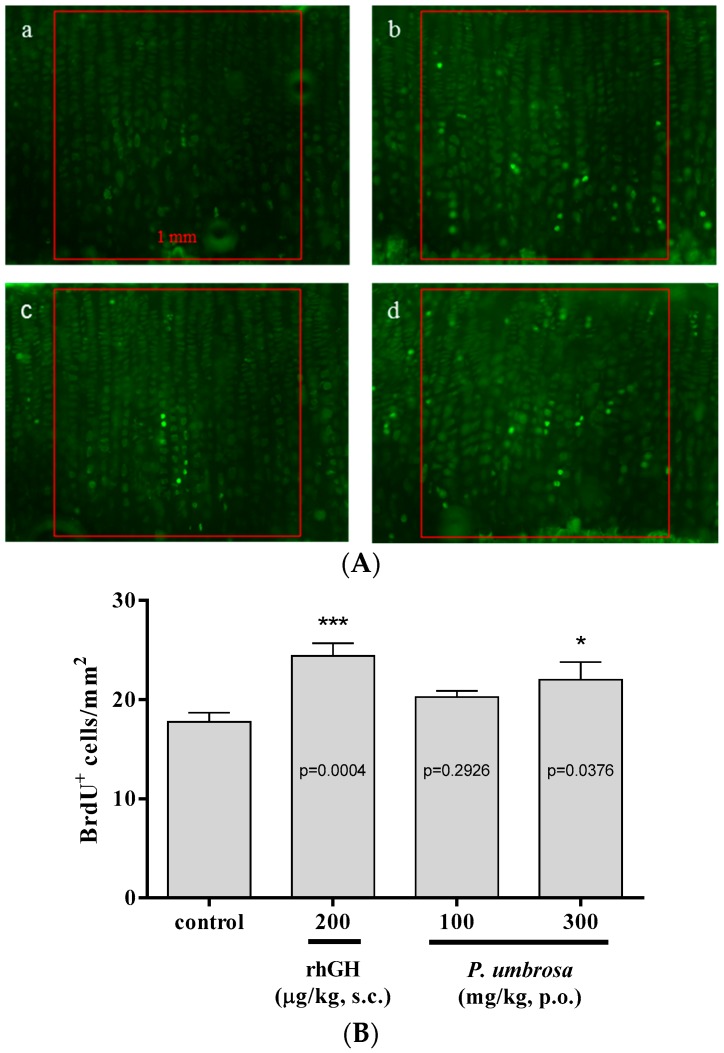
(**A**) Representative photomicrographs of 5-bromo-2′-deoxyuridine (BrdU)-labeled chondrocytes of the proximal tibial growth plate, Green: BrdU-labeled chondrocyte, (**a**) vehicle treated control group; (**b**) rhGH 200 μg/kg (s.c.) treated group; (**c**) *P. umbrosa* 100 mg/kg (p.o.) treated group; (**d**) *P. umbrosa* 300 mg/kg (p.o.) treated group. Each box area is 1 mm^2^; (**B**) Effects of *P. umbrosa* on chondrocyte proliferation of proximal tibial growth plate in female adolescent rats. Each value is the mean ± SD. The number of animals is four per group; * *p* < 0.05 and *** *p* < 0.001 *vs.* control (one-way ANOVA, Bonferroni test).

BrdU-labeled cells were observed to assess chondrocytes proliferation in the proximal tibial growth plate (Figure 4A). The number of BrdU-labeled chondrocytes of the growth plate in the control group was 17.8 ± 3.3 cells/mm^2^ and in the rhGH group was 24.4 ± 4.4 cells/mm^2^. BrdU-labeled chondrocytes in the *P. umbrosa* at doses of 100 and 300 mg/kg group also significantly increased, to 20.3 ± 2.2 cells/mm^2^ and 22.0 ± 6.2 cells/mm^2^, respectively (Figure 4B).

### 2.5. Effects on IGF-1 and BMP-2 Expression

**Figure 5:**
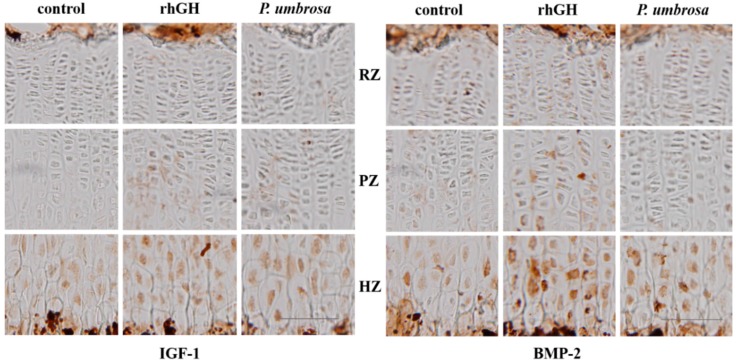
Immunohistochemical localization of insulin-like growth factor-1 and bone morphogenetic protein-2 on the proximal tibial growth plate in rats, control: vehicle treated control group, rhGH: rhGH 200 μg/kg (s.c.) treated group, *P. umbrosa*: *P. umbrosa* 300 mg/kg (p.o.) treated group, RZ: resting zone, PZ: proliferative zone, HZ: hypertrophic zone. The scale bar is 100 μm.

Protein expression of IGF-1 and BMP-2 in the proximal tibial growth plate was assessed with antigen-specific immunohistochemistry. IGF-1 was more highly expressed in hypertrophic zones than in resting and proliferative zones. Administration of *P. umbrosa* or rhGH remarkably increased the intensity of IGF-1 expression in proliferative and hypertrophic zone compared with control. BMP-2 expression was also higher in hypertrophic zones and markedly increased by administration of *P. umbrosa* or rhGH particularly (Figure 5).

### 2.6. Effect on Serum IGF-1 and IGFBP-3 Concentrations

**Figure 6:**
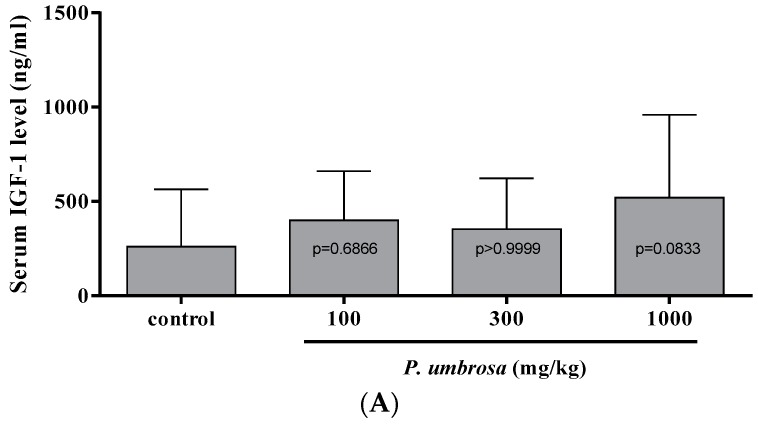

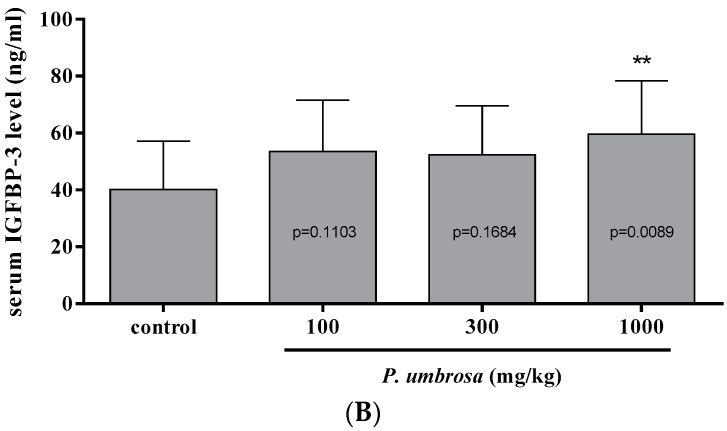
(**A**) Serum insulin-like growth factor-1 concentration for each group at 12 h after treatment; (**B**) Serum insulin-like growth factor binding protein-3 concentration for each group at 12 h after treatment. Each value is the mean ± SD. The number of animals is sixteen per group; ** *p* < 0.01 *vs.* control (one-way ANOVA, Bonferroni test).

To evaluate the effect of *P. umbrosa* on serum IGF-1 and IGFBP-3 concentrations, serum concentrations were measured by specific ELISA at 12 h after treatment, which is reported as the highest point of serum IGF-1 and IGFBP-3 concentration after single injection of rhGH in rats [38]. *P. umbrosa* at doses of 100, 300 and 1000 mg/kg showed a dose-dependent increment of serum IGFBP-3 level that reached 48.4% at 1000 mg/kg compared to the control group. Serum IGF-1 level was slightly higher at every dose level but not significantly different compared to the control group (Figure 6).

## 3. Discussion

Oral administration of 70% EtOH extracts of *P. umbrosa* significantly increased longitudinal bone growth rate, height of hypertrophic zone, and chondrocyte proliferation of the proximal tibial growth plate compared to control. *P. umbrosa* also increased serum IGFBP-3 levels and upregulated the expressions of IGF-1 and BMP-2 in hypertrophic zone of the growth plate.

*P. umbrosa* called *Xu Duan*, which literally means “reconnect what is broken”, was used to treat bone fractures in traditional medicine. Bone fracture recovery involves a process of endochondral ossification by proliferation and differentiation of chondrocytes and osteoblasts, which mimics the process of longitudinal bone growth [25]. Moreover, the dichloromethane subfraction of *P. umbrosa* extracts has been reported to increase new bone formation of calvarial defects in rats and increase collagen synthesis and alkaline phosphatase activity in primary cultured osteoblast derived from alveolar bone [39,40]. These mechanisms might be shared with the effects of *P. umbrosa* on the stimulation of longitudinal bone growth rate.

*P. umbrosa* at doses of 100 and 300 mg/kg was shown to increase the bone growth rate to 380.9 and 381.5 μm/day in the proximal tibial growth plate, respectively. The bone growth rate in the control group was 358.2 μm/day in accordance with the results reported previously [41]. Tetracycline gets deposited in newly formed bones, causing a fluorescent line corresponding to the injection, and the distance between the chondro-osseous junction and the fluorescent line indicates the growth rate, which is the length of bone growth during a certain period of time [37]. The result suggests that *P. umbrosa* increases longitudinal bone growth rate.

*P. umbrosa* at dose of 300 mg/kg significantly increased overall height of growth plate by 12.8% especially that of hypertrophic zone by 13.5% compared to the control group in the proximal tibial growth plate. Growth plate consists of chondrocytes with three distinctive histological areas: resting, proliferative and hypertrophic zones [18]. Rapid chondrocyte division in the proliferative zone and substantial chondrocyte enlargement in the hypertrophic zone lead to increase in bone growth rate and growth plate height [42]. Especially, the greatest contribution to the overall height of growth plate increment is the increase in hypertrophic zone height which reflects the rapid longitudinal bone growth rate [24,43]. Previous studies have found good correlations between bone growth rate and the height of hypertrophic zone, regardless of bone location or animal age [44,45,46]. The result suggests that *P. umbrosa* increases the height of growth plate, especially that of the hypertrophic zone.

Because the bone growth rate is equal to the product of the rate of proliferation and the size of hypertrophic chondrocyte, the proliferation of chondrocytes in the growth plate is a critical determinant of the increase in body length [42]. In the present study, proliferation of chondrocyte was determined experimentally by counting labeled nuclei by BrdU, a thymidine analog that incorporates into newly synthesized DNA in proliferating cells during the S-phase of cycle [47]. The number of BrdU-labeled cells in *P. umbrosa* group significantly increased compared to the control group, suggesting that *P. umbrosa* increases proliferation rate of chondrocytes in the growth plate.

*P. umbrosa* increased the expressions of local IGF-1 and BMP-2 in the proliferative and hypertrophic zones of the tibial growth plate. Bone growth is the outcome of chondrocyte proliferation and hypertrophy of the growth plates caused by either circulating IGF-1 secreted from the liver by GH or local IGF-1 production by direct stimulation of GH. Local IGF-1 production is mainly dependent on serum GH and is combined with IGF-1 receptor expressed on the cell surface of the chondrocytes in the growth plate, like systemic IGF-1 [17]. Locally expressed IGF-1 in the growth plate is the primary mediator of the direct effects of GH on the longitudinal bone growth rate [48,49]. Regarding other factors affecting GH, BMP-2 expressed in the growth plate has been reported to accelerate the bone growth by stimulating proliferation and hypertrophy of chondrocytes in an organ culture model [50]. Recently, it has been reported that circulating GH increases local production of BMP-2 in the growth plate while circulating IGF-1 does not affect its local production [51]. These findings suggest that GH-dependent increase of BMP-2 production in the growth plate may be attributable to direct growth-promoting effects of GH. Taken together, the results suggest that growth stimulating effects of *P. umbrosa* might be attributable to local IGF-1 and BMP-2 expression stimulated by the direct effect of GH on the local growth plate. 

Oral administration of *P. umbrosa* at doses of 100, 300 and 1000 mg/kg at 12 h before sacrifice, showed dose-dependent increment of serum IGFBP-3 level that reached 48.4% at 1000 mg/kg compared to the control group. Serum IGF-1 level was slightly higher at every dose level but not significantly different compared to the control group. This result concurs with other studies showing that the level of IGFBP-3 may be superior to the measurement of IGF-1 in the diagnosis of GH deficiency [52,53], and in reflecting actual serum levels of IGF-1 [54]. It is well known that IGF-1 transport is mediated mainly by IGFBP-3 [55] and IGFBP-3 is considered as a biochemically excellent index of GH level because it is GH dependent and is maintained at a regular daily concentration [56]. Our results suggest that the effect of *P. umbrosa* on longitudinal bone growth rate may also be mediated by the increment in serum IGF-1 and IGFBP-3 concentrations stimulated by GH.

In summary, *P. umbrosa* increases the longitudinal bone growth rate accompanying promotion of chondrocyte proliferation and differentiation, with the increment of circulating IGFBP-3. Regarding the immunohistochemical study, the effect of *P. umbrosa* may also be attributable to upregulating local IGF-1 and BMP-2 expressions in the growth plate, which can be considered as normal functioning of GH dependent autocrine/paracrine pathway. Based on these findings, it is tempting to speculate that *P. umbrosa* may be a therapeutic candidate for the children with short stature.

## 4. Experimental Section

### 4.1. Plant Material

The root of *P. umbrosa* was purchased from the Zhashui Livelihood and Welfare Company (Shaanxi, China). *P. umbrosa* was identified by Dr Hocheol Kim, Department of Herbal Pharmacology, Kyung Hee University, where a voucher specimen (#HP125) was deposited on 29 January 2015.

### 4.2. Sample Preparation and HPLC Analysis

The dried roots of *P. umbrosa* were extracted in a reflux apparatus (70% aqueous ethanol, 6 h at 80 °C). The filtered extract was lyophilized after concentration under reduced pressure. The yield of extract was 20.3%. The quantitative analysis of *P. umbrosa* performed by HPLC. The HPLC system consisted of a 1525 pump (Waters, Milford, MA, USA), a 2707 autosampler and a Waters 2998 PDA. Separation was achieved on Waters Sunfire™ C^18^ (250 mm × 4.6 mm i.d., 5 μm) column at 40 °C. The mobile phase composition was 0.5% H_3_PO_4_ (A) and acetonitrile (B) eluted for separation as following: 0–20 min, 5%–17%; 20–30 min, 17%–22%; 30–40 min, 22%–30% solvent B. The flow rate was 1.0 mL/min. The injection volume was 5 μL and effluent was monitored at 235 nm. The extract was analysed in triplicate. Shanzhiside methyl ester was chosen as a marker compound to standardize the extract because of its characteristic peak and good stability in aqueous solution.

### 4.3. Animals

Twenty-five-day-old female Sprague-Dawley rats were obtained from Samtako (Osan, Korea). This study was done in accordance with the guidelines of the Institutional Animal Care and Use Committee of Kyung Hee University (KHUASP[SE]-13-028). All animals were housed under controlled conditions (22 ± 1 °C, 12 h of light starting at 07:00) in an isolated ventilated chamber with food and water available *ad libitum*.

### 4.4. Treatment

After 7 days of acclimatization, rats were divided into four groups: control, *P. umbrosa* 100 mg/kg, *P. umbrosa* 300 mg/kg and recombinant human GH (rhGH) 200 μg/kg. Vehicle, *P. umbrosa* 100 mg/kg or *P. umbrosa* 300 mg/kg were orally administered twice daily (8:30 a.m.; 8:30 p.m.) and rhGH 200 μg/kg (Eutropin, LG Life Sciences, Seoul, Korea) was subcutaneously injected once daily (8:30 a.m.) for 10 consecutive days. On the 11th day, rats were sacrificed for analysis.

### 4.5. Longitudinal Bone Growth Rate

Tetracycline hydrochloride was injected intraperitoneally 72 h prior to sacrifice (20 mg/kg, Sigma Aldrich, St. Louis, MO, USA). Tibias were dissected, fixed with 4% paraformaldehyde, decalcified in 50 mM ethylenediaminetetraacetic acid solution (Sigma Aldrich). After dehydration in 30% sucrose, the samples were cut at sagittal sections of the proximal part with thickness of 40 μm using a cryostat (CM3050S, Leica Microsystems, Berlin, Germany). Bone growth rate per day was assessed by measuring distance between the chondro-osseous junction within the growth plate and the proximal endpoint of the tetracycline label, and dividing the distance into three. Fluorescent line was viewed with an epifluorescence microscope (BX50, Olympus, Tokyo, Japan) and the distance was blind read with Image J software (NIH, Bethesda, MD, USA) by three different researchers to avoid the possible distinction among individuals.

### 4.6. Growth Plate Height

Cresyl violet (CV, Sigma Aldrich) staining of the chondrocytes was used to measure growth plate height. The heights of the overall growth plate, resting, proliferative and hypertrophic zone were measured at three different locations by using Image J software. The proliferative zone (PZ) was measured from flat chondrocytes aligned with the long axis of the bone presumed to be proliferative. The hypertrophic zone (HZ) was measured from chondrocyte with swollen nuclei and cytoplasm, which is easily distinguished based on size. The resting zone (RZ) was measured by subtraction of the height of proliferative and hypertrophic zones from the overall height of growth plate.

### 4.7. Chondrocyte Proliferation

To label S-phase nuclei for studies of chondrocyte proliferation, rats were injected intraperitoneally with BrdU (50 mg/kg, Sigma Aldrich) on days 8, 9 and 10. Dehydrated sagittal sections were pretreated as described previously [6] and reacted with BrdU-specific mouse antibody diluted 1/100 overnight at 4 °C (Santa Cruz Biotechnology, Santa Cruz, CA, USA). Sections were washed and reacted with FITC mouse antibody diluted 1/200 for 4 h (Jackson Immunoresearch Laboratories, West Grove, PA, USA). The chondrocyte proliferation was measured by the number of BrdU-labeled cells per unit area (mm^2^) in the proximal tibial growth plate.

### 4.8. Immunohistochemistry

To detect IGF-1 and BMP-2 expression in the growth plate, dehydrated sagittal sections of tibia were pretreated as described previously [9] and reacted with rabbit IGF-1 antibody and goat BMP-2 primary antibody diluted 1/200 overnight (Santa Cruz). The sections were washed, reacted with biotinylated rabbit antibody diluted 1/200 (Jackson Immunoresearch Laboratories) and incubated with avidin-biotin complex reagent diluted 1/100 (Vectastain ABC Kit, Vector Laboratories, Burlingame, CA, USA) for 1 h, respectively. Sections were developed with 0.05% 3,3-diaminobenzidine (Sigma Aldrich) solution containing hydrogen peroxide.

### 4.9. Serum IGF-1 and IGFBP-3

Thirty-three-day-old female Sprague–Dawley rats were obtained from Samtako and acclimatized for 7 days prior to the oral administration. Blood was collected from the jugular vein at 12 h after single administration of *P. umbrosa* at doses of 100, 300, and 1000 mg/kg. Serum IGF-1 and IGFBP-3 were measured by specific ELISA kits according to the manufacturer’s protocols (Biovendor, Modrice, Czech Republic).

### 4.10. Statistical Analysis

Statistical analyses were performed using GraphPad Prism 6 software (GraphPad Software, La Jolla, CA, USA). One-way analysis of variance (ANOVA) with *post-hoc* Bonferroni test for multiple comparisons. statistical significance was accepted at *p* < 0.05 when the Bonferroni test was applied. All values were presented as mean ± SD.

## Figures and Tables

**Table 1:** Zonal height of each group in growth plate of proximal tibia in rats.

Height (μm)	Control	rhGH 200 μg/kg (s.c.)	*P. umbrosa* 300 mg/kg (p.o.)
Overall growth plate	453.8 ± 28.6	536.9 ± 29.5 ***	508.6 ± 28.9 **
Resting zone	27.3 ± 3.5	25.1 ± 2.9	29.8 ± 2.5
Proliferative zone	111.7 ± 23.1	110.0 ± 30.0	120.9 ± 5.9
Hypertrophic zone	304.0 ± 46.1	384.0 ± 54.2 **	345.0 ± 22.0 *

Data are shown as mean ± SD. The number of animals is eight per group; * *p* < 0.05, ** *p* < 0.01 and *** *p* < 0.001 *vs.* control.
